# Comparison between STIR and T2-weighted SPAIR sequences in the evaluation of inflammatory sacroiliitis: diagnostic performance and signal-to-noise ratio

**DOI:** 10.1590/0100-3984.2019.0077

**Published:** 2020

**Authors:** Vitor Faeda Dalto, Rodrigo Luppino Assad, Mario Müller Lorenzato, Michel Daoud Crema, Paulo Louzada-Junior, Marcello Henrique Nogueira-Barbosa

**Affiliations:** 1 Faculdade de Medicina de Ribeirão Preto da Universidade de São Paulo (FMRP-USP), Ribeirão Preto, SP, Brazil.; 2 Radiologia Especializada de Ribeirão Preto, Ribeirão Preto, SP, Brazil.; 3 Institut National du Sport, de l’Expertise et de la Performance (INSEP), Paris, France.

**Keywords:** Sacroiliitis, Sacroiliac joint, Magnetic resonance imaging, Spondyloarthropathies, Signal-to-noise ratio, Sacroiliite, Articulação sacroilíaca, Ressonância magnética, Espondiloartropatias, Relação sinal/ruído

## Abstract

**Objective:**

To compare two different fat-saturated magnetic resonance imaging (MRI) techniques-STIR and T2 SPAIR-in terms of image quality, as well as in terms of their diagnostic performance in detecting sacroiliac joints (SIJ) active inflammation.

**Materials and Methods:**

We included 69 consecutive patients with suspected spondyloarthritis undergoing MRI between 2012 and 2014. The signal-to-noise ratio (SNR) was calculated with the method recommended by the American College of Radiology. Two readers evaluated SIJ MRI following ASAS criteria to assess diagnostic performance regarding the detection of active SIJ inflammation. T1 SPIR Gd+ sequence was used as the reference standard.

**Results:**

The mean SNR was 72.8 for the T1 SPIR Gd+ sequence, compared with 14.1 and 37.6 for the STIR and T2 SPAIR sequences, respectively. The sensitivity and specificity of STIR and SPAIR T2 sequences did not show any statistically significant differences, for the diagnosis of sacroiliitis with active inflammation.

**Conclusion:**

Our results corroborate those in the recent literature suggesting that STIR sequences are not superior to T2 SPAIR sequences for SIJ evaluation in patients with suspected spondyloarthritis. On 1.5-T MRI, T2-weighted SPAIR sequences provide better SNRs than do STIR sequences, which reinforces that T2 SPAIR sequences may be an advantageous option for the evaluation of sacroiliitis.

## Abstract (pt)

**Objetivo:**

O objetivo deste estudo foi comparar a qualidade da imagem das técnicas de saturação de gordura T2 SPAIR e STIR e, adicionalmente, comparar o desempenho diagnóstico das duas sequências para detecção de inflamação ativa nas imagens de ressonância magnética (RM) das sacroilíacas.

**Materiais e Métodos:**

Foram incluídos 69 pacientes consecutivos que realizaram RM das articulações sacroilíacas no período de 2012 a 2014. O cálculo da relação sinal/ruído (RSR) foi realizado pelo método do Colégio Americano de Radiologia. O desempenho diagnóstico foi realizado utilizando os resultados da leitura das imagens da sequência T1 SPIR Gd+ como referência.

**Resultados:**

A avaliação da RSR mostrou média de 72,8 para a sequência T1 SPIR Gd+. A sensibilidade e a especificidade das sequências STIR e SPAIR 2 para o diagnóstico de sacroiliite com inflamação ativa não apresentaram diferenças estatisticamente significantes.

**Conclusão:**

Nossos resultados reforçam a literatura recente sugerindo que não há superioridade da sequência STIR em relação à sequência SPAIR T2 para a avaliação das sacroilíacas em pacientes com espondiloartrite. A sequência SPAIR T2 apresenta melhor RSR em relação à sequência STIR em imagens de RM de 1,5 T, o que reforça que a sequência SPAIR T2 possa ser utilizada como opção vantajosa na avaliação da sacroiliite inflamatória.

## INTRODUCTION

Early diagnosis is essential for the treatment of axial spondyloarthritis^(1)^, and magnetic resonance imaging (MRI) is the recommended imaging method because it is able to identify findings corresponding to the phase of active inflammation, before the appearance of radiographic findings^(1,2)^. On MRI, active inflammation is characterized by the presence of subchondral bone marrow edema in fluid-sensitive sequences or by contrast enhancement of bone marrow^(2)^.

Previously, the Assessment of SpondyloArthritis International Society (ASAS) MRI working group recommended the use of short-tau inversion-recovery (STIR) sequences to identify the bone marrow edema that is indicative of active inflammation of the subchondral tissue^(3)^. More recently, the ASAS MRI working group began to recommend the use of T2-weighted fluid-sensitive sequences, such as STIR sequences, as well as T2-weighted sequences with fat suppression, for that purpose^(4)^.

In 2017, two different groups of researchers compared STIR sequences with other fluid-sensitive sequences in terms of their ability to detect active inflammation of the sacroiliac joints and found no superiority of the STIR sequences over T2-weighted spectral attenuated inversion-recovery (T2 SPAIR) sequences and T2-weighted Dixon sequences, respectively^(5,6)^.

The signal-to-noise ratio (SNR) is an important parameter in the objective assessment of MRI image quality^(7)^, being one of the parameters used in the American College of Radiology (ACR) MRI accreditation program^(8)^. The SNR is a measure of the relationship between the pure signal coming from the studied tissue, which is the signal we truly want to detect, and the background noise. A higher SNR corresponds to a lower effect of background noise on signal detection or measurement. A lower SNR results in an image with a grainy aspect that makes it difficult to analyze the original signal.

To our knowledge, there have been no studies comparing the SNR of STIR sequences with that of T2 SPAIR sequences in the evaluation of the sacroiliac joints in 1.5-T MRI scanners. Therefore, the objective of this study was to perform a comparative analysis of T2 SPAIR and STIR sequences in terms of the SNR in the sacroiliac region and in terms of their diagnostic performance in the detection of active inflammation of the sacroiliac joints.

## MATERIALS AND METHODS

This was a retrospective, cross-sectional study in which we included consecutive patients undergoing MRI of the sacroiliac joints at our institution between 2012 and 2014. We included patients between 18 to 65 years of age who were under clinical suspicion of having spondyloarthritis. A total of 69 patients were considered eligible. The images were evaluated in accordance with the ASAS criteria for the diagnosis of axial spondyloarthritis. We also calculated sensitivity and specificity. The study was approved by the local research ethics committee (Ref. 30607314.9.0000.5440). Because of the retrospective nature of the study, the requirement for written informed consent was waived.

The images were anonymized and evaluated with the open-source software Horos, version 2.1.1 (The Horos Project) to determine the SNR. Because the sample size calculation indicated that at least 30 examinations would be necessary in order to carry out the SNR assessment with adequate statistical power, we selected the MRI examinations of 60 patients. In ten of those examinations, the field of view did not include the air space necessary to perform the reading, all of those examinations consequently being excluded from the assessment. Therefore, the final sample comprised 50 MRI examinations of patients under clinical suspicion of having spondyloarthritis.

**Figure 1:**
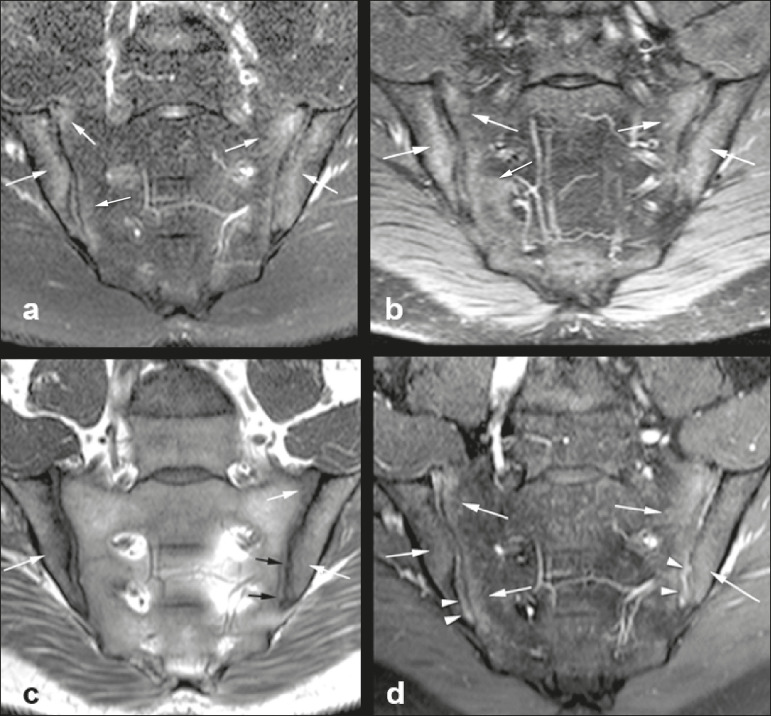
Male, 42 years old, diagnosed with ankylosing spondylitis. Coronal oblique MRI images. SPAIR T2 (**a**), STIR (**b**), T1 weighted without fat suppression (**c**), and SPIR T1 Gd + (**d**) sequences. The white arrows indicate areas of subchondral bone edema in both sacroiliac joints involving the iliac and sacral portions. The white arrowheads indicate intra-articular enhancement in the SPIR T1 Gd + sequence. The black arrows indicate areas of erosion on the iliac margin of the left sacroiliac.

All MRI examinations were performed in a 1.5-T scanner (Achieva; Philips Medical Systems, Best, The Netherlands), with a 17-channel spine coil and the patients in the supine position. The protocol employed for evaluating sacroiliitis includes the following: T1-weighted coronal sequences; coronal STIR sequences; coronal T2 SPAIR sequences; and T1-weighted gadolinium-enhanced spectral presaturation with inversion recovery (T1 SPIR Gd+) sequences. The acquisition parameters for the sequences are shown in Table 1. The mean total acquisition time per patient ranged from 22 min to 25 min. The images acquired are similar to that illustrated in Figure 1.

**Table 1:** MRI pulse sequences and acquisition parameters for the evaluation of the sacroiliac joints.

Coronal T2 SPAIR
FOV: 160 × 160 × 70 mm (LL × AP × CC); slice thickness: 4 mm; TE: 60 ms; TR: 1500-3000 ms; voxel size: 1.07 × 1.19 mm; matrix: 48 × 127; acquisition time: 4 min.
Coronal T1-weighted
FOV: 160 × 160 × 70 mm (LL × AP × CC); slice thickness: 4 mm; TE: 8.5 ms; TR: 400-700 ms; voxel size: 0.9 × 1.25 mm; matrix: 176 × 127; acquisition time: 2 min 14 s.
Coronal STIR
FOV: 160 × 302 × 62 mm (LL × AP × CC); slice thickness: 4 mm; TE: 10 ms; TR: 2000-3000 ms; TI: 170 ms; voxel size: 0.5 × 1.25 mm; matrix: 332 × 242; acquisition time: 4 min 40 s.
Coronal T1 SPIR Gd+
FOV: 160 × 160 × 92 mm (LL × AP × CC); slice thickness: 4 mm; TE: 10 ms; TR: 550-600 ms; voxel size: 0,9 × 1,25 mm; matrix: 168 × 120; acquisition time: 4 min.

LL, laterolateral; AP, anteroposterior; CC, craniocaudal.

The SNR was calculated by using the method recommended by the ACR^(8)^. In brief, the SNR is obtained by dividing the mean signal for a region of interest (ROI), designated the signal ROI, by the standard deviation obtained in another ROI positioned over the signal-free air space, designated the background ROI.

To measure the signal, the ACR recommends the use of an ROI of at least 50 pixels, positioned over the tissue of interest. Because radiologists evaluate spondyloarthritis by looking for changes in the subchondral bone marrow, it was decided that the signal ROI should be positioned over the bone marrow. As a reference standard for subchondral bone marrow evaluation, we chose to position the signal ROI over the central region of the sacrum. The placement of the signal ROI over the subchondral bone of the sacroiliac joint would be subject to variations related to patient pathological status (illness or health), although determining that status was not within the scope of this study, which was focused on the SNR of the sequences, regardless of the presence or absence of disease.

**Figure 2:**
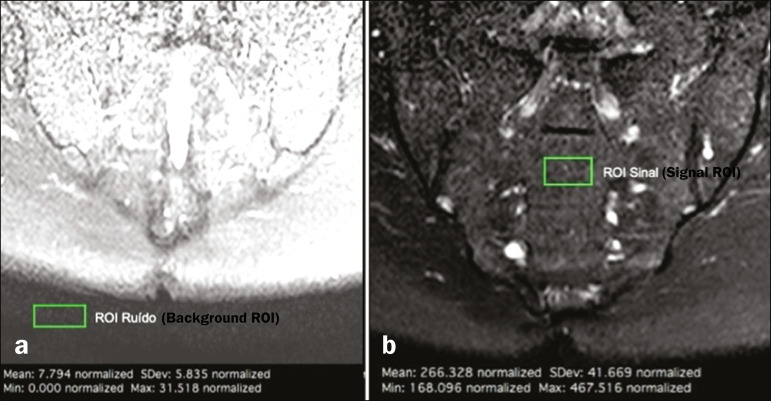
Acquisition of signal and noise data in the Horos program, in order to determine the SNR. **a:** The image shows the collection of background noise from the background ROI, positioned in the air. **b:** The image shows the collection of the mean bone marrow signal from the signal ROI, positioned over the central region of the sacrum.

To obtain the background noise value, the ACR suggests the creation of an ROI in the external air space surrounding the patient, in an area free of artifacts. In the 50 patients evaluated here, an air ROI with a minimum of 300 pixels (a background ROI) was selected for each sequence, the Horos software being used in order to collect the standard deviation from the background ROI (Figure 2).

The SNR was obtained by dividing the mean signal of the signal ROI by the standard deviation of the background ROI. That calculation was performed for all three MRI sequences (T2 SPAIR, STIR, and T1 SPIR Gd+) in each the 50 patients separately, and the data were subsequently placed in a table for statistical evaluation.

Two musculoskeletal radiologists with 15 years and 4 years of experience in evaluating MRI scans of the musculoskeletal system, respectively, evaluated each sequence and classified the examination as positive or negative for sacroiliitis, according to the diagnostic criteria of the ASAS working group^(2)^. According to the ASAS criteria, examinations showing subchondral bone edema in two consecutive images or at two different sites within the sacroiliac joints are considered positive for inflammatory sacroiliitis.

In the assessment of diagnostic performance, the results of the reading of the T1 SPIR Gd+ sequences were used as a reference. The positivity or negativity for inflammatory activity in the T1 SPIR Gd+ sequence was determined by consensus between the two readers. In cases of disagreement, a third radiologist, with 14 years of experience in interpreting MRI scans of the musculoskeletal system, made the final decision.

### Statistical analysis

In the evaluation of the SNR, the correlation between sequences was performed by paired t-tests, the differences in SNRs identified for each sequence in each patient serving as the dependent variables. Sensitivity and specificity measurements were performed for each reader and for each study sequence (STIR and T2 SPAIR) in comparison with the gold standard (T1 SPIR Gd+). The level of interobserver agreement was determined by calculating the kappa statistic. Values of *p* < 0.05 were considered statistically significant.

## RESULTS

**Figure 3:**
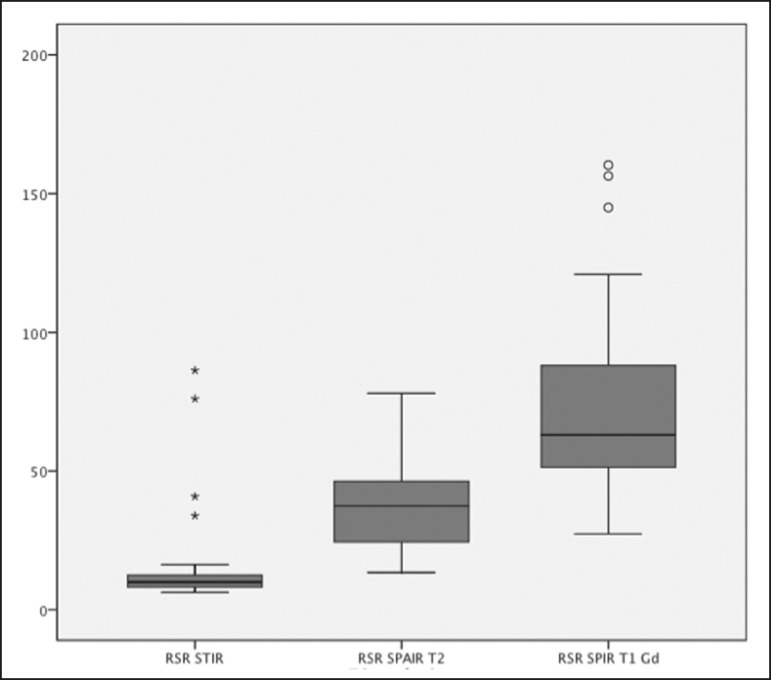
Box plot of the distribution of SNR values for the STIR, T2 SPAIR, and T1 SPIR Gd+ sequences. Note the high signal intensity in the T1 SPIR Gd+ sequences and the higher mean SNR for the T2 SPAIR sequences than for the STIR sequences.

In the evaluation of image quality, the mean SNR was 72.8 for the T1 SPIR Gd+ sequence, 37.6 for the T2 SPAIR sequence, and 14.1 for the STIR sequence. The distribution of the SNR values can be seen in Figure 3.

The comparative evaluation of the mean SNRs between the sequences, performed in a paired manner, patient by patient, by the paired t-test, revealed a difference of 58.7 (95% CI: 68.0-49.4) between T1 SPIR Gd+ and STIR, the mean SNR being significantly higher for the T1 SPIR Gd+ sequence (*p* < 0.000). Comparing the signal of the T2 SPAIR sequence with that of the T1 SPIR Gd+ sequence, we found a difference between the two mean SNRs of 35.2 (95% CI: 42.4-28.1), the mean SNR also being significantly higher for the T1 SPIR Gd+ sequence (*p* < 0.000). The difference in the mean SNR between the STIR and T2 SPAIR sequences was 23.4 (95% CI: 17.2-29.6), the mean SNR being significantly higher for the T2 SPAIR sequence (*p* < 0.000). The diagnostic performance of the T2 SPAIR and STIR sequences is shown in Table 2.

**Table 2:** Sensitivity (95% CI), specificity (95% CI), positive predictive value, negative predictive value, and accuracy, by reader, for the T1 STIR Gd+ and T2 SPAIR sequences.

	Sensitivity	Specificity	PPV	NPV	Accuracy
T1 STIR Gd+					
Reader 1	0.94 (0.69-0.99)	0.87 (0.73-0.93)	0.69	0.97	0.88
Reader 2	0.94 (0.69-0.99)	0.90 (0.78-0.96)	0.76	0.97	0.91
T2 SPAIR					
Reader 1	1.00 (0.80-1.00)	0.94 (0.84-0.98)	0.85	1.00	0.96
Reader 2	0.94 (0.69-0.99)	0.94 (0.83-0.98)	0.84	0.98	0.94

PPV, positive predictive value; NPV, negative predictive value.

## DISCUSSION

In the present study, we identified a statistically significant difference in SNR between the STIR and T2 SPAIR sequences, the SNR being better for the latter. The SNR was highest for the T1 SPIR Gd+ sequence, followed by the T2 SPAIR sequence, being lowest for the STIR sequence. In the qualitative evaluation of diagnostic performance, we found no significant difference between the STIR and T2 SPAIR sequences in terms of sensitivity, specificity, accuracy, negative predictive value, or positive predictive value for the diagnosis of active inflammatory sacroiliitis, in accordance with the ASAS working group diagnostic criteria and using the T1 SPIR Gd+ sequence as the reference.

Recent studies have shown that the STIR and T2 SPAIR sequences were comparable on the basis of the Spondyloarthritis Research Consortium of Canada (SPARCC) score^(5,9)^. Our results are consistent with those findings, although we used a different method from that employed in one of those studies^(5)^, comparing the two methods quantitatively in relation to the SNR and performing a qualitative analysis of their diagnostic performance. In that study, conducted by Dalto et al.^(5)^, the diagnostic performance was assessed by extrapolating from the SPARCC score, whereas in our study it was calculated directly using the ASAS working group criteria for active inflammation. Dalto et al.^(5)^ also assessed interobserver agreement among specialists in radiology and rheumatology, whereas our readers were musculoskeletal radiologists. Greese et al.^(10)^ conducted a study comparing T2-weighted fat-saturated turbo spin-echo sequences with proton-density STIR sequences in a 3.0-T scanner and found that the former had a better SNR and was better able to identify bone edema. Although those authors reported results similar to ours for SNR, they studied the SNR in images of the sacroiliac joints acquired in 3.0-T scanners, whereas the images evaluated in our study were acquired in 1.5-T scanners. Nevertheless, the mean SNRs reported by those authors were similar to those obtained in the present study.

The identification of sacroiliac joints inflammation is of great importance for the diagnosis of axial spondyloarthritis. The relevant findings in the evaluation of the sacroiliac joints include enthesitis, synovitis, capsulitis, and erosions^(11)^. The STIR sequence is just one of the many possible methods of fat suppression, which makes it possible to identify bone edema^(12,13)^. Currently, there are several MRI fat-saturation techniques available. The various techniques are based on the frequency of precession in the electron cloud surrounding the hydrogen proton, which is different in water than in fat. That difference makes it possible to create techniques based on the chemical shift. For fat saturation, there are techniques based on chemical shift, such as chemical-shift selective saturation, water excitation, and Dixon techniques; techniques based on inversion, such as STIR; and hybrid techniques, such as SPAIR and SPIR^(14,15)^.

Since the STIR sequence was first described by Bydder et al.^(16)^, the technique has been widely used. It is not very sensitive to field heterogeneity and has good fat saturation, even outside the central region of the magnetic field. It also has satisfactory fat saturation, even in the presence of metal implants. The disadvantage of the STIR sequence is its low SNR. That disadvantage could be mitigated by shortening the echo time, although that also reduces the T2 weighting of the image^(14)^.

Hybrid sequences, such as SPAIR, combine an inversion-recovery pulse with an adiabatic radiofrequency pulse. Studies have shown that SPAIR sequences are relatively insensitive to field heterogeneity and tend to have better SNRs than do STIR sequences^(14)^. In contrast, the Dixon techniques have low sensitivity to field heterogeneity and excellent SNRs, even in areas near metallic implants, those advantages being most pronounced in the three-point water-fat decomposition method^(17)^.

The image quality assessment performed in the present study also confirmed previous findings showing that the SNR is higher for SPAIR sequences than for STIR sequences^(4,9,13)^. In our study, the T2 SPAIR sequence showed a mean SNR 2.66 times greater than that of the STIR sequence and the mean examination time was comparable between the two. This can be considered the greatest advantage of the T2 SPAIR sequence and one of the reasons for its use. Even when using similar examination times, slice thickness, and T2-weighting parameters, we found a higher SNR for the T2 SPAIR sequence. The SPAIR sequence has more options with respect to slice thickness, T2 weighting, and matrix, without substantially impairing the image quality, because it has a higher SNR^(7)^.

Our study has some limitations, including those inherent to its retrospective design. Another limitation is that the T1 SPIR Gd+ sequence with fat suppression was used as a reference to calculate the diagnostic performance of the STIR and T2 SPAIR sequences. Although subchondral tissue biopsy would probably be the ideal reference, it would be unethical to subject patients to an invasive procedure in order to validate a concept of comparing MRI sequences. Therefore, we decided to use the reference that has been most widely employed in the evaluation of active inflammation of the sacroiliac joints^(5,18,19)^.

Our findings support those reported recently in the literature, suggesting that there is no superiority of the STIR sequence over other fluid-sensitive sequences for the evaluation of subchondral bone edema of the sacroiliac joints in patients with spondyloarthritis^(5,6,10)^. We found no difference between the STIR and T2 SPAIR sequences in terms of their diagnostic performance in identifying active inflammation of the subchondral bone tissue of the sacroiliac joints. Our results also indicate that, on 1.5-T sacroiliac joints MRI, the T2 SPAIR sequence provides a better SNR than does the STIR sequence, which reinforce that the use of T2 SPAIR sequences is a viable option for the evaluation of inflammatory sacroiliitis, with some advantage over STIR sequence.
